# Nitrated α–Synuclein Immunity Accelerates Degeneration of Nigral Dopaminergic Neurons

**DOI:** 10.1371/journal.pone.0001376

**Published:** 2008-01-02

**Authors:** Eric J. Benner, Rebecca Banerjee, Ashley D. Reynolds, Simon Sherman, Vladimir M. Pisarev, Vladislav Tsiperson, Craig Nemachek, Pawel Ciborowski, Serge Przedborski, R. Lee Mosley, Howard E. Gendelman

**Affiliations:** 1 Center for Neurovirology and Neurodegenerative Disorders, Department of Pharmacology and Experimental Neuroscience, University of Nebraska Medical Center, Omaha, Nebraska, United States of America; 2 Department of Pathology and Microbiology, University of Nebraska Medical Center, Omaha, Nebraska, United States of America; 3 Department of Internal Medicine, University of Nebraska Medical Center, Omaha, Nebraska, United States of America; 4 Nebraska Informatics Center for the Life Sciences, Eppley Cancer Institute, University of Nebraska Medical Center, Omaha, Nebraska, United States of America; 5 Department of Neurology, and Cell Biology and The Center for Motor Neuron Biology and Disease, Columbia University, New York, New York, United States of America; 6 Department of Pathology and Cell Biology and The Center for Motor Neuron Biology and Disease, Columbia University, New York, New York, United States of America; Swiss Federal Institute of Technology Lausanne, Switzerland

## Abstract

**Background:**

The neuropathology of Parkinson's disease (PD) includes loss of dopaminergic neurons in the substantia nigra, nitrated α-synuclein (N-α-Syn) enriched intraneuronal inclusions or Lewy bodies and neuroinflammation. While the contribution of innate microglial inflammatory activities to disease are known, evidence for how adaptive immune mechanisms may affect the course of PD remains obscure. We reasoned that PD-associated oxidative protein modifications create novel antigenic epitopes capable of peripheral adaptive T cell responses that could affect nigrostriatal degeneration.

**Methods and Findings:**

Nitrotyrosine (NT)-modified α-Syn was detected readily in cervical lymph nodes (CLN) from 1-methyl-4-phenyl-1,2,3,6-tetrahydropyridine (MPTP) intoxicated mice. Antigen-presenting cells within the CLN showed increased surface expression of major histocompatibility complex class II, initiating the molecular machinery necessary for efficient antigen presentation. MPTP-treated mice produced antibodies to native and nitrated α-Syn. Mice immunized with the NT-modified C-terminal tail fragment of α-Syn, but not native protein, generated robust T cell proliferative and pro-inflammatory secretory responses specific only for the modified antigen. T cells generated against the nitrated epitope do not respond to the unmodified protein. Mice deficient in T and B lymphocytes were resistant to MPTP-induced neurodegeneration. Transfer of T cells from mice immunized with N-α-Syn led to a robust neuroinflammatory response with accelerated dopaminergic cell loss.

**Conclusions:**

These data show that NT modifications within α-Syn, can bypass or break immunological tolerance and activate peripheral leukocytes in draining lymphoid tissue. A novel mechanism for disease is made in that NT modifications in α-Syn induce adaptive immune responses that exacerbate PD pathobiology. These results have implications for both the pathogenesis and treatment of this disabling neurodegenerative disease.

## Introduction

Parkinson's disease (PD) is a common progressive neurodegenerative disease clinically characterized by resting tremor, muscle rigidity, bradykinesia, and postural instability [1]. PD is sporadic and of unknown cause although host genetics, environmental cues, aging, impaired energy metabolism and oxidative stress are linked to disease onset and progression [2]. Pathologically, PD is characterized by degeneration of dopaminergic cell bodies in the substantia nigra pars compacta (SNpc) and their associated caudate projections [1]. Nonetheless, the pathological hallmark of PD is cytoplasmic inclusions of fibrillar, misfolded proteins called Lewy bodies composed principally of α-synuclein (α-Syn) [3].

α-Syn is a 140-amino acid (aa), natively unfolded, soluble protein that is localized in the pre-synaptic terminals of neurons of the central nervous system (CNS), where it interacts with and may regulate synaptic vesicles [3], [4], [5], [6], [7], [8]. Three missense mutations (A53T, A30P and E46K) in the gene encoding α-Syn are linked to dominantly inherited PD [9], [10], [11]. Moreover, multiplication of the wild-type (WT) gene has also been linked to PD, suggesting that the level of α-Syn is an important pathogenic factor [12], [13]. Such familial cases are rare and in sporadic PD, there is no genetic aberration of α-Syn. However, it has been proposed that post-translational modifications such as nitration enhances WT α-Syn propensity to aggregate [14], [15], [16], [17]. Oxidized and aggregated α-Syn, when released from dying neurons, may stimulate scavenger receptors on microglia resulting in their sustained activation and dopaminergic neurodegeneration [18], [19], [20]. Moreover, activated microglia generate nitric oxide and superoxide that rapidly react to form peroxynitrite [21] which can then traverse cell membranes resulting in 3-nitrotyrosine (NT) formation, DNA damage, mitochondrial inhibition, or lipid peroxidation [22].

We now propose that modified “self” epitopes as neo-epitopes, including NT modifications within α-Syn, can bypass or possibly break immunological tolerance [23], [24], [25], [26], [27], [28], [29] and activate peripheral leukocytes in draining lymphoid tissue. In keeping with this, NT-modifications incorporated into self-peptides were sufficient to evade immunological tolerance as was previously reported [30]. The recruitment of activated T cells, specific for disease-associated protein modifications in α-Syn, may, in turn, promote a toxic microglial phenotype. The role of the adaptive immune system is becoming increasingly important in “non-autoimmune” diseases of the CNS [31]. Research in traumatic and neurodegenerative models have suggested a neuroprotective role for T and B cells within the CNS and that manipulation of the peripheral immune system can affect neurodegeneration [32]. Our own studies demonstrated that immunization of mice with glatiramer acetate generate T cells that recognize myelin basic protein (T_MBP_), secrete interleukin (IL)-10, IL-4, and transforming growth factor (TGF)-β, and confer protection against 1-methyl-4-phenyl-1,2,3,6-tetrahydropyridine (MPTP)-induced neurodegeneration presumably by suppression of microglial activation [33]. Antibodies generated through active immunization of human α-Syn transgenic mice with purified human α-Syn protein reduced α-Syn aggregation in cell bodies and terminals, and was associated with protection of dopaminergic nerve terminals [34]. The conclusions were that anti-α-Syn antibodies target the aggregated protein to lysosomal pathways for degradation and that the strategy could be applied for treatment of human disease. That work was conducted however in an animal model of PD that lacks a neuroinflammatory component. As such, the study did not address the cellular arm of the immune system, which likely requires cytokine and chemokine gradients for efficient cell entry into diseased regions. Nevertheless, the work of Masliah et al., 2005 supports the potential importance for adaptive immunity and for immune-based strategies for the treatment of PD. Certainly, research activities into the potential use of α-Syn as an immunogen will require further study.

Here, we report that NT-modified CNS antigens drain to the deep cervical lymph nodes (CLN) of mice following exposure to MPTP. Moreover, antigen-presenting cells (APC) within CLN increase surface expression of major histocompatibility complex (MHC) class II, initiating the molecular machinery necessary for efficient antigen presentation. The differential outcome on the susceptibility to MPTP-induced dopaminergic neurodegeneration amongst WT and severe combined immunodeficient (SCID) mice suggest a functional link of the adaptive immune system to MPTP-induced neurotoxicity. We further demonstrate in mice of two disparate haplotypes, that adoptive transfer of T cells from syngeneic WT donors immunized with nitrated α-Syn (N-α-Syn) prolongs MPTP-induced dopaminergic neuronal loss and hence warrants caution against the use of N-α-Syn or self-proteins that are prone to nitrate modifications for vaccine-based PD therapies.

## Materials and Methods

### Animals

Male 6–7 week old, WT C57BL/6J (stock 000664, denoted as B6) (H-2^b^), B6.CB17-*Prkdc^scid^*/SzJ (stock 001913, herein denoted as SCID) (H-2^b^) and B10.BR-*H2^k^ H2-T18^a^*/SgSnJ (stock 000465, herein denoted as B10.BR) (H-2^k^) mice were purchased from Jackson Laboratories (Bar Harbor, ME). All animal procedures were in accordance with National Institutes of Health (NIH) guidelines and approved by the Institutional Animal Care and Use Committee (IACUC) of the University of Nebraska Medical Center (UNMC).

### MPTP Intoxication

For chronic intoxication, B6 mice received 5 intraperitoneal (i.p.) injections at 24 hr intervals for 5 days of either vehicle (PBS, 10 ml/kg) or MPTP-HCl (30 mg/kg of free base in PBS) (Sigma-Aldrich, St. Louis, MO). For acute intoxication, mice received 4 i.p. injections, one every 2 hr, of either vehicle (PBS, 10 ml/kg) or MPTP-HCl (18 mg/kg of free base in PBS for B10.BR mice, 14 or 18 mg/kg for B6 mice). At selected time points following MPTP intoxication, mice were sacrificed and brains processed for subsequent analyses. MPTP handling and safety measures were in accordance with published guidelines [35].

### Immunohistochemistry

At the time points indicated following MPTP intoxication, mice were transcardially perfused with 4% paraformaldehyde (PFA) in 0.1 M PBS using 0.9% saline as vascular rinse. Brains were post-fixed in 4% PFA overnight, kept in 30% sucrose for 2 days, snap frozen, embedded in OCT compound, and 30 µm sections cut on a cryostat (CM1900, Leica, Bannockburn, IL). The sections were collected in PBS and processed free-floating. Primary antibodies used for immunohistochemistry includes rabbit anti-TH antibody (1∶2000; Calbiochem/EMD Biosciences, Inc., San Diego, CA), rat anti-mouse CD11b or Mac-1 (1∶1,000; Serotec, Raleigh, NC), rat anti-CD3 (1∶800; BD Pharmingen, San Diego, CA,), rat anti-CD4 (BD Pharmingen), and rat anti-CD8 (BD Pharmingen). Immunostaining was visualized using diaminobenzidine (Sigma-Aldrich) as the chromogen and mounted on slides. TH, CD3-, CD4- and CD8-immunostained brain sections were counterstained with thionin (Sigma-Aldrich) as previously described [33], [36]. Fluoro-Jade C (Chemicon International, Inc., Temecula, CA) was used to stain degenerating neurons [37] and was detected as green fluorescence by fluorescence microscopy with FITC filter (Eclipse E800, Nikon, Inc., Melville, NY).

### Stereology of TH-Positive Neurons

Total numbers of Nissl- and TH-stained neurons throughout the entire SNpc were counted stereologically in a blinded fashion with Stereo Investigator software (MicroBrightfield, Williston, VT) using the Optical Fractionator probe module as previously described [33].

### Cloning α-Syn and 4YSyn

Total RNA from adult C57BL/6 mouse brain was extracted using TRIzol reagent (Invitrogen, Carlsbad, CA) according to the manufacturer's instructions. The full-length mouse α-Syn gene (504 bp) and 120 bp length encoding the C-terminal portion (4YSyn) was amplified by reverse transcriptase-polymerase chain reaction (RT-PCR) using Platinum Taq DNA Polymerase High Fidelity (Invitrogen). The 5′ primer was designed to introduce a Nde1 site at position 1. This fragment was blunt cloned into the pZero-1 (Invitrogen) Eco RV site using standard cloning procedures. Transformed cells were plated on low salt agar containing Zeocyn and 3 mM IPTG. Colonies were screened using colony PCR with α-Syn primers. Colonies containing the full-length mouse α-Syn gene or the 3′ fragment encoding 4YSyn were grown overnight and plasmid DNA was isolated using standard mini-prep (Invitrogen). The gene was digested out of pZero with Nde1 and Xho1 and subcloned into the Nde1 and Xho1 sites in the pET-28a prokaryotic expression vector using DH5-α cells. Colonies were screened using colony PCR. Purified plasmids were submitted to the UNMC core facility for sequence confirmation. Plasmids containing the complete sequence were transformed into BL-21 *E. coli* cells for expression. Frozen glycerol stocks were maintained at −80°C.

### 4YSyn Expression

Glycerol stocks were streaked on Luria-Bertani (LB) agar plate containing 30 µg/ml kanamycin. A single colony was inoculated into LB broth containing 30 µg/ml kanamycin, grown for 8 hrs, and stored at 4°C until the following day. The starter culture was diluted 1∶100 into fresh liquid medium containing 30 µg/ml kanamycin and allowed to grow to an OD_600_ = 0.6. Expression of recombinant protein was induced by the addition of 3 mM IPTG with continued incubation for 3 hrs at 37°C. Following induction, cells were centrifuged, weighed, and stored at −80°C until purification protocol was resumed. >90% of detectable 4YSyn was found in the soluble fraction.

### Protein Purification and Nitration

Cell lysis was performed with Bug Buster reagent (Novagen/EMD Biosciences, Inc., San Diego, CA) at 5 ml/g cells with addition of EDTA-free protease inhibitor cocktail (Calbiochem). Benzonase nuclease (Novagen) was added to reduce viscosity during lysis following manufacturer's instructions. Insoluble cell debris was removed by centrifugation at 16,000×g for 20 min at 4°C. The soluble fraction was directly subjected to column affinity chromatography and was carried out in the following steps: His-tagged protein was bound to Ni-NTA His Bind Resin (Novagen) in Bug Buster reagent with the addition of imidazole (10 mM). The column was washed first with 50 mM NaH_2_PO_4_/300 mM NaCl/20mM imidazole, pH 8.0 and then with 50 mM NaH_2_PO_4_/300 mM NaCl/35mM imidazole, pH 8.0. Elution was carried out 50 mM NaH_2_PO_4_/300 mM NaCl/250 mM imidazole pH 8.0. Samples were separated by SDS-PAGE and stained with Brilliant Blue G-Colloidal Coomassie stain (Invitrogen) to confirm purity of the eluted fraction. Full-length α-Syn and 4YSyn were visualized by silver stain (Silver Xpress, Invitrogen). Purified full-length α-Syn was dialyzed in 50 mM NaH_2_PO_4_ buffer. Thrombin cleavage was carried out using biotinylated thrombin cleavage capture kit (Novagen) following manufacturer's instructions. Cleaved His-tags were removed with Ni-NTA resin. His-tag free full-length α-Syn and His-tagged 4YSyn (unable to remove the His-tag) were dialyzed against water for 24–48 hrs with multiple water changes, lyophilized, and weighed. Endotoxin was removed by polymyxin B agarose beads following manufacturer's instructions (Sigma-Aldrich) and tested for residual endotoxin by Limulus amebocyte lysate (LAL) assay (E-Toxate, Sigma-Aldrich). Recombinant α-Syn-derived proteins were endotoxin-free as all batches of purified proteins utilized tested below the limit of detection for endotoxin by LAL (<0.05 endotoxin units, EU).

Lyophilized protein was resuspended (2 mg protein/ml) in 50 mM NaH_2_PO_4_ buffer containing 5 mM FeCl_3_ as a Lewis acid. Peroxynitrite (Upstate Biotechnology, Inc. Lake Placid, NY) was added dropwise to protein to achieve a 5 M excess while vigorously mixing the reaction mixture. Nitrated protein was dialyzed against water for 48 hrs using multiple water exchanges, lyophilized, and stored at −80°C.

### MALDI-TOF Mass Spectrometry

MALDI-TOF mass spectrometric analysis was performed using a Voyager DE Pro mass analyzer (Applied Biosystems, Framingham, MA), which was externally calibrated prior to each assay. Data acquisition was performed using 500 laser shots. The MS scan range was set from 500 to 20,000 m/z. Saturated α-cyanohydroxycinnamic acid (Sigma-Aldrich) was used as matrix in these assays and samples were manually spotted onto MALDI targets.

### Enzyme-Linked Immunosorbent Assay (ELISA)

Individual wells of Immunolon II ELISA plates (Thermo Electron Corp., Waltham, MA) were coated with 100 µl/well of native 4YSyn or N-4YSyn at 1 µg/ml PBS, pH 8.5. Plates were incubated for 2 hrs at 37°C and washed with 0.5% Tween20/PBS, pH 7.2 (PBS-T). Nonspecific binding was blocked by the addition of 1% bovine serum albumin in PBS, pH 7.2 (PBS-BSA) and incubation at 37°C for 1 hr. Plates were washed with PBS-T, 100 µl of 2-fold serial dilutions (from an initial 1∶50 dilution in PBS-BSA) of serum samples from MPTP- or PBS-treated mice were added to each well, and incubated at 37°C for 1 hr. Plates were washed with PBS-T and 100 µl/well of a 1∶5000 dilution of horseradish peroxidase (HRP)-conjugated anti-mouse IgG (SouthernBiotech, Birmingham, AL) was added. Plates were incubated at 37°C for 1 hr, washed with PBS-T, and reacted with 0.012% H_2_O_2_ and 2.2 mM *o*-phenylenediamine dihydrochloride (Sigma-Aldrich) in 100 µl of 0.1 M phosphate-citrate buffer, pH 5.0. The reaction was stopped with the addition of 2 N H_2_SO_4_, read at 490 nm on a microplate reader (Vmax Kinetic Microplate Reader, Molecular Devices Corporation, Sunnyvale, CA), and acquired data analyzed with interfacing SoftMax Pro software (Molecular Devices). Serum IgG concentrations were quantified from a standard curve prepared from known concentrations of mouse IgG (SouthernBiotech).

### Immunization and Immune Cell Adoptive Transfers

B10.BR (H-2^K^) mice were immunized with PBS, 50 µg of 4YSyn or N-4YSyn emulsified in an equal volume of CFA containing 1 mg/ml *Mycobacterium tuberculosis* (Sigma-Aldrich). B6 (H-2^b^) mice were immunized with PBS, 10 µg of 4YSyn or N-4YSyn with or without CFA. While immunization with adjuvant were administered subcutaneous (s.c.) on either side of the tail base, s.c. injections without adjuvant were given at 5 different sites. Fourteen days after primary immunization, mice were boosted with their respective antigens. CFA recipient mice were boosted with their respective antigens emulsified in IFA (Sigma-Aldrich). Five days following their final immunizations, donor mice were sacrificed and single cell suspensions were prepared from the spleen and draining lymph nodes after lysing red blood cells with ammonium chloride-potassium (ACK) lysis buffer (0.15M NH_4_Cl, 10 mM KHCO_3_, 0.1 mM Na_2_EDTA, pH7.2). T cells were enriched by using the PAN T cell isolation kit (Miltenyi Biotec, Auburn, CA) and by depletion of magnetically labeled cells employing AutoMACS (Miltenyi Biotec). Twelve hrs post-MPTP intoxication, both B10.BR and B6 mice received intravenous (i.v.) injections of 5×10^7^ spleen cells (SPC) in 0.25 ml of Hanks' balanced salt solution (HBSS). B10.BR mice also received 2.5×10^7^ purified T cells. SCID mice were reconstituted with i.v. injections of 8×10^7^ unfractionated SPC populations from WT B6 mice. RCS-SCID mice were rested for 4 wks prior to MPTP intoxication.

### ^3^H-Thymidine *in vitro* Proliferation Assays

Samples of pooled immunized donor cells used for adoptive transfer were tested for their proliferative capacity by ^3^H-thymidine incorporation after stimulation with either immunizing or irrelevant antigen. Donor SPC were plated at a density of 2×10^6^ cells/ml complete RPMI tissue culture media [RPMI 1640 supplemented with 10% fetal bovine serum (FBS), 2 mM L-glutamine, 10 mM HEPES, 1 mM sodium pyruvate, 1× nonessential aa, 55 µM 2-mercaptoethanol, 100 U/ml penicillin, and 100 µg/ml streptomycin (Mediatech Inc., Herndon, VA)]. SPC from PBS, 4YSyn, and N-4YSyn immunized mice were stimulated with 0, 1, 10, 50 µg/ml of immunizing antigen, 4YSyn or N-4YSyn, and cultured at 37°C for 5 days. Cells were pulsed with 1 µCi ^3^H-thymidine/well for the final 18 hrs of culture, harvested onto glass fiber plates, and counted by β-scintillation spectrometry (TopCount, Packard-PerkinElmer Instruments, Wellesley, MA).

### Western Blot Analysis

Ventral midbrain (VMB) and lymphoid organ protein extracts (80 µg/lane) were separated by 16% SDS-PAGE (Invitrogen) and transferred for 45 min onto 0.2 µm PVDF membranes (Millipore, Bedford, MA). Membranes were probed with rabbit antibodies to NT (1∶2000; Chemicon) or monoclonal rat antibodies to myelin basic protein (MBP, 1∶1000, Chemicon) or guinea pig antibodies to α-Syn (1∶1000; Ab-1, Oncogene/EMD Biosciences). Appropriate HRP-conjugated secondary antibodies (Santa Cruz Biotechnology, Santa Cruz, CA) were used to visualize blots using SuperSignal West Pico Chemiluminescent substrate and CCL-XPosure film (Pierce Biotechnology, Inc., Rockford, IL). Immunoblots were stripped and reprobed with antibodies to α-actin (1∶5000; Chemicon,) as an internal control.

### Identification of α-Syn in MPTP-CLN

Anti-N-α/β-syn (clone nSyn12, mouse ascites, Upstate) that specifically recognizes N-α-Syn (14.5 kD) and N-β-Syn (17 kD) but not non-nitrated α/β-Syn was used for immunoprecipitation (IP). VMB and CLN from PBS or MPTP treated mice were dissected out, homogenized in ice-cold RIPA buffer, pH 7.4 and centrifuged at 10,000×g for 10 min at 4°C to remove cellular debris. Protein A/G PLUS-Agarose beads (Santa Cruz Biotechnology) were added to 2 mg total cellular protein, incubated for 1 hr at 4°C. Beads were centrifuged at 1,000×g for 5 min at 4°C. The supernatant was incubated with 40 µl anti-N-α-Syn overnight at 4°C on a rotating device, and then with Protein A/G PLUS-Agarose beads for 1 hr on a rotating device at 4°C. Immunoprecipitates were collected after centrifugation at 1,000×g for 5 min at 4°C, washed once with RIPA buffer and twice with PBS, resuspended in 40 µl of 1× electrophoresis sample buffer.

N-α-Syn IP samples were fractionated by large format 16% Tricine SDS-PAGE (Jule Inc., Milford, CT; BIORAD Laboratories, Inc, Los Angeles, CA) at constant voltage for 8–10 hrs. The gel was stained with highly sensitive SYPRO Ruby stain (Invitrogen) and scanned at excitation (400 nm) and emission (630 nm) wavelengths using Typhoon scanner (Amersham Biosciences, Piscataway, NJ) to visualize the protein bands. Small gel fragments (3–4 mm) corresponding to molecular weight (12–18 kD) were excised from each lane of the same gel stained with Coomassie. In brief, gel pieces were destained for 1 hr at room temperature using 100 µl of 50% ACN/50 mM NH_4_CO_3_. Gel pieces were dried and incubated with trypsin in 10 mM NH_4_CO_3_ (Promega, Madison, WI) overnight at 37°C. Peptides were extracted by washing gel pieces twice with 0.1% TFA and 60% ACN. Dried samples were resuspended in 12 µl of 0.1% formic acid in water for automated injection. All samples were purified using ZipTip (Millipore) prior to MS analysis. In-gel trypsin digested proteins were fractionated on microcapillary RP-C18 (Ciborowski et al., 2004). The resulting peptides were sequenced using Electrospray Ionization (ESI)-LC MS/MS (Proteome X System with LCQDecaPlus mass spectrometer, thermoElectron, Inc., San Jose, CA) with a nanospray configuration. The spectra obtained from LC-MS/MS analysis were searched against the NCBI.fasta rodent protein database using SEQUEST search engine (BioWorks 3.2 SR software from ThermoElectron, Inc, San Jose, CA.). Criteria for high confidence protein identification were used as previously published [38], [39], [40], [41], [42], [43].

### Flow Cytometry

Single cell suspensions were prepared from deep CLN from C57BL/6 mice 20–24 hrs post PBS or MPTP (18 mg/kg) intoxication. Cell suspensions were analyzed for cell surface expression of CD11c, CD11b, and MHC class II (I-A^b^). Also, prior to adoptive transfers, cell populations from immunized donors were stained for T cells using PE conjugated anti-mouse CD3ε (BD Pharmingen) and B cells with FITC conjugated anti-mouse CD19 (BD Pharmingen). Analysis was performed with a FACSCalibur flow cytometer interfaced with CellQuest software (BD-Biosciences, Immunocytometry Systems, San Jose, CA).

### Determination of N-4YSyn-mediated Toxicity *In Vitro*

For proliferation analyses, purified T cells from naïve B6 mice were plated with SPC irradiated at 3000 rad (1:3) at 2×10^6^ cells/ml in complete RPMI tissue culture media and activated with anti-CD3 (0.5 µg/ml, 145-2C11; BD Pharmingen) in U-bottom 96-well tissue culture plates. Graded concentrations of 4YSyn or N-4YSyn were added to quadruplicate wells. After activation for 3 days, ^3^H-thymidine incorporation was performed as described previously.

To assess α-Syn-mediated cytotoxicity, purified T cells were stimulated with anti-CD3 and cultured at a density of 1×10^6^ cells/ml for 24 hrs in media alone or in the presence of 4YSyn or N-4YSyn at concentrations of 1, 3, 10, or 30 µg/ml. Cells were stained with PI, washed and the percentages of PI^+^ dead cells and MFI were analyzed by flow cytometry.

### Macrophage and MES 23.5 Cultures

BMM were prepared from C57BL/6 adult male (6–12 weeks old) mice. The animals were sacrificed by CO_2_ asphyxiation. Single cell suspensions of bone marrow cells were obtained from femur bone marrow cavities after flushing with HBSS, and red blood cells lysed with ACK buffer. The bone marrow cells were cultured in complete DMEM medium (Dulbecco's Modified Eagles Media supplemented with 10% FBS, 2 mM L-glutamine, and 1% penicillin/streptomycin) containing 2 µg/ml macrophage colony stimulating factor (MCSF), a generous gift from Wyeth Pharmaceuticals (Cambridge, MA) in a 5% CO_2_/37°C incubator. Non-adherent cells were removed from flasks at 1, 4, and 7 days by successive DMEM washes. Adherent BMM were harvested and replated for experiments following 7–14 days of culture. Cells from the MES 23.5 dopaminergic cell line kindly provided by Dr. Stanley Appel, were cultured in 75-cm^2^ flasks in DMEM/F12 with 15 mM HEPES (Invitrogen) containing N2 supplement (Invitrogen), 100 U/ml of penicillin, 100 µg/ml streptomycin, and 5% FBS. Cells were grown to 80% confluence then co-cultured with BMM in serum free MEM/F12 at a density of 1×10^5^ cells (1:1) on sterile glass coverslips.

### N-α-Syn SPC-Induced Microglia Cytotoxicity

SPC isolated from N-4YSyn (10 µg) immunized B6 mice were cultured in RPMI media and activated *in vitro* for 4 days with N-4YSyn (1 µg/ml). MES 23.5 cells or macrophages alone or MES 23.5 and macrophage co-cultures were stimulated with aggregated N-α-Syn (1.45 µg/ml) alone and in combination with either activated SPC or supernatants obtained from activated SPC for 24 hrs. Unstimulated cultures served as controls. Assays for viable and dead cells were performed with Live/Dead Viability/Cytotoxicity kit (Invitrogen) according to the manufacturer's protocol and viewed under a fluorescence microscope (Nikon Eclipse E800, Buffalo Grove, IL). Images were captured at 100× magnification and quantification of live (green) and dead (red) counts was performed from 4–8 different fields.

### Cytokine array

Triplicate co-cultures of antigen presenting cells (APC) and T cells from PBS-, 4YSyn- and N-4YSyn-immunized mice were stimulated with 4YSyn or N-4YSyn. After 24 hours of culture, 50 µl samples were collected, centrifuged, and supernatants frozen at −80°C until utilized. Frozen supernatants were thawed only once and analyzed using the BD Cytometric Bead Array Mouse Th1/Th2 Kit (BD Biosciences, San Jose, CA) according to the manufacturer's instructions.

### Statistical Analysis

All values are expressed as mean±SEM. Differences among normally distributed means were evaluated by Student's *t* test for two group comparisons or one-way ANOVA followed by Bonferroni post-hoc tests for pairwise comparisons amongst multiple data sets (Statistica v7, Statsoft, Tulsa, OK, and SPSS v13, SPSS, Inc., Chicago, IL) and were considered significant at p ≤ 0.05 unless otherwise indicated. Kolmogorov-Smirnov (K-S) analysis was performed for flow cytometric data analysis.

## Results

### CNS Antigens Drain to CLN Following MPTP Intoxication

**Figure 1:**
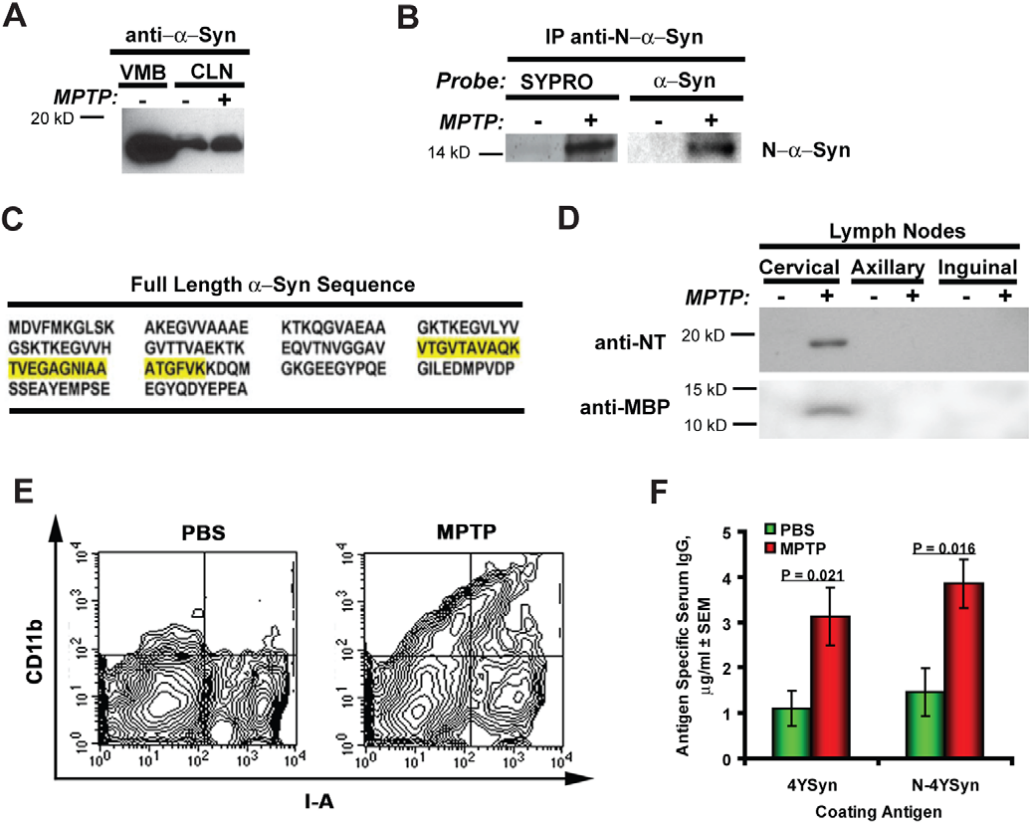
Drainage of N-α-Syn and MBP to CLN with macrophage activation and production of α-Syn serum antibodies after MPTP intoxication. (A) Western blot of tissue homogenates from VMB and CLN of mice 20 hrs following treatment with PBS or MPTP, were probed with antibodies to α-Syn. (B) N-α/β Syn IP with (clone nSyn12 antibodies) against CLN homogenates from PBS or MPTP-treated mice. Immunoprecipitates were fractionated on a 16% polyacrylamide gel and the gel stained with SYPRO Red or blotted. The Western blot was probed with anti-α-Syn. (C) Proteins recovered from in-gel digestion of 12–18 kD fragments from anti-N-α/β Syn of CLN immunoprecipitates were were identified by LC-MS/MS. The sequence coverage by peptides identified by LC-MS/MS from the CLN of MPTP-treated mice is highlighted in yellow within the primary aa sequence of full-length mouse α-Syn. (D) Western blots of lymph node homogenates (Cervical, Axillary, and Inquinal) from mice treated with PBS or MPTP. Blots were probed with antibodies to nitrotyrosine (NT) or anti-myelin basic protein (MBP). (E) Flow cytometric analysis of CD11b and I-A^b^ expression in cells from CLN, show an increased number of CD11b^+^I-A^+^ cells 24 hrs after MPTP treatment compared to PBS administered animals (n = 3 mice/group). (F) Antibodies against α-Syn and N-α-Syn in sera of B6 WT mice on day 21 following MPTP intoxication (n = 8) or PBS control treatment (n = 5) as determined by anti-α-Syn specific ELISA. Sera from MPTP treated group contained significantly higher IgG antibodies directed against 4YSyn (p = 0.021) and N-4YSyn (p = 0.016) compared to PBS treated control sera. Comparisons of mean IgG concentrations ± SEM was by Student's *t* test.

To determine if CNS antigens drain to CLN during established neurodegeneration of the nigrostriatal pathway, their presence in ventral midbrain (VMB), cervical, axillary, inguinal, mesenteric lymph nodes and spleens were determined 24 hrs after MPTP-intoxication in C57BL6 mice. The presence of unmodified α-Syn was demonstrated in VMB and CLN (Figure 1A) as well as other lymph nodes and in the spleen from phosphate-buffered (PBS)- or MPTP-treated mice in anti-α-Syn-probed immunoblots (data not shown). These findings also confirmed the expression of unmodified α-Syn amongst cells of hematopoietic lineage [44]. N-α-Syn IP showed that N-α-Syn was present in the CLN of MPTP-treated, but not PBS-treated mice as similar molecular weight bands were observed from gels probed with SYPRO Red and Western blots performed with α-Syn antibodies (Figure 1B). To validate the presence of NT-modified α-Syn after MPTP treatment, N-α-Syn immunoprecipitates were obtained after in-gel tryptic digestion of 12–18 kD fragments acquired from the VMB and CLN and sequenced by LC-MS/MS. This regions was chosen as it represents the molecular mass ranges of oxidized α-Syn [6], [14], [45], [46], [47]. Sequence analysis demonstrated α-Syn peptides (yellow highlighted sequences, Figure 1C) in the VMB from both PBS- and MPTP-treated mice but exclusively in the CLN of MPTP-intoxicated mice (Table 1). α-Syn peptides were identified at >99.999% confidence (Table 1). Western blot analysis of lymphoid tissue homogenates using rabbit NT antibodies detected a single band with a molecular mass of ∼16–18 kD–which is comparable to that of α-Syn–in CLN from MPTP-intoxicated animals, but not in other lymph nodes or spleen (Figure 1D, blots of mesenteric lymph nodes and spleen are not shown). These results were confirmatory for the presence of N-α-Syn in the draining CLN. Another CNS antigen, MBP was also detected only in the CLN of MPTP intoxicated animals (Figure 1D). NT-modified proteins and MBP were absent in lymph nodes and spleens of control (PBS-injected) mice. These data suggest that brain proteins released as a consequence of nigrostriatal injury, drain to the deep CLN, placing them in organs associated with efficient presentation of antigen. To demonstrate the functional significance of these observations, single cell suspensions were prepared from CLN isolated from MPTP animals and controls, and analyzed by flow cytometry for MHC class II expression on CD11b^+^ APC (Figure 1E). Increased frequencies of CD11b^+^/MHC class II^+^ in MPTP-treated mice compared to PBS controls was taken as evidence of leukocyte activation in the deep CLN following MPTP-induced nigrostriatal injury. Supporting the induction of a α-Syn specific immune response, sera from WT B6 mice 21 days after chronic MPTP intoxication were analyzed for anti-α-Syn IgG and compared to animals that received PBS. Serum levels of α-Syn antibodies in mice exposed to MPTP were significantly increased (Figure 1F). Together, these results demonstrate that NT-modified α-Syn draining into the deep CLN is capable of eliciting a peripheral immune response.

**Table 1:** Probabilities (p values) of protein sequence matches within 12–18 kD bands from anti-N-α/β-synuclein immunoprecipitation and LC MS-MS analyses of VMB and CLN from PBS- or MPTP-treated mice.

Protein match	P value for protein matches from:			
	VMB		CLN	
	PBS	MPTP	PBS	MPTP
α-synuclein	5.9×10^−7^	7.7×10^−6^		1.0×10^−6^
β-synuclein	4.3×10^−7^	3.8×10^−9^		
myelin basic protein		3.4×10^−5^		
MHC class I antigen				4.0×10^−3^
immunoglobulin heavy chain variable region		5.8×10^−5^		
chemokine-like factor super family five variant 4	3.8×10^−4^			
ribosomal protein S14			5.6×10^−6^	
Tesp4 protein			1.8×10^−5^	
A chain A, complex of the second kunitz domain of tissue factor pathway inhibit		2.0×10^−4^		8.7×10^−5^
structural constituent of ribosome				6.3×10^−5^
parotid secretory protein			2.6×10^−4^	
ribosomal protein S14				4.9×10^−4^
Similar to NADH dehydrogenase (ubiquinone) 1 beta subcomple	5.9×10^−4^			
mediator of RNA polymerase II transcription, subunit 8 homolog isoform				9.9×10^−4^
cAMP-dependent protein kinase, alpha-catalytic subunit (PKA C-alpha)			1.3×10^−3^	
step II splicing factor SLU7			2.4×10^−3^	
parotid secretory protein				3.5×10^−3^
Similar to protease, serine, 3	1.2×10^−5^			
hypothetical protein LOC320696			4.5×10^−3^	
Unknown (protein for MGC:116262)				2.9×10^−6^
unnamed protein product (16288 kD)				1.6×10^−4^
unnamed protein product (25311 kD)			1.1×10^−5^	
unnamed protein product (27163 kD)		2.4×10^−4^		
unnamed protein product (58621 kD)	1.2×10^−5^			8.2×10^−5^
unnamed protein product (65626 kD)	2.7×10^−6^	1.4×10^−5^		2.5×10^−5^
pancreatic trypsin 1	3.7×10^−5^	2.7×10^−6^	1.5×10^−6^	1.3×10^−4^
trypsin 10	2.9×10^−3^	1.8×10^−4^	5.1×10^−5^	8.6×10^−5^
trypsinogen 7		2.2×10^−5^		2.6×10^−6^

### Adaptive Immunity Participates in MPTP-Nigral Degeneration

**Figure 2:**
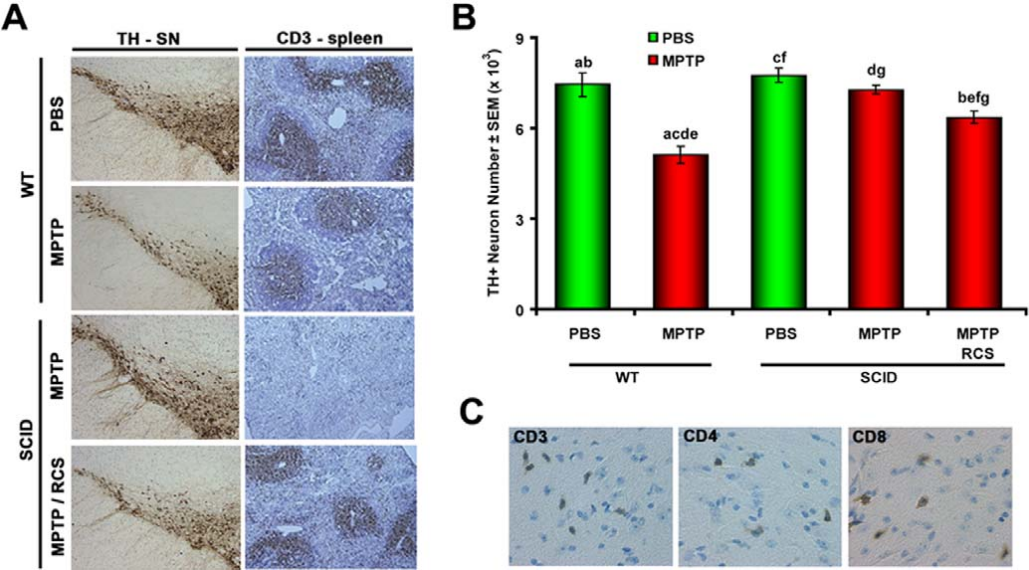
Nigral degeneration following MPTP-intoxication in B6 SCID mice before and after lymphoid cell reconstitution. (A) Photomicrographs of TH-immunostained SN (left panels) and CD3-immunostained spleen sections (right panels) from B6 (WT), SCID, and reconstituted SCID (RCS-SCID) mice treated with PBS or MPTP and obtained on day 21 post-MPTP intoxication. Immunostaining for expression of CD3 in spleens show normal distributions of CD3^+^ T cells in B6 WT and RCS-SCID mice treated with PBS or MPTP. Note the absence of CD3^+^ T cells in spleens from SCID MPTP mice. (B) Quantification of TH+ neurons in the SN of B6 WT, SCID, or reconstituted (RCS) SCID mice treated with PBS or MPTP. Values represent mean number of TH+ neurons ± SEM for 5-9 mice per group. ^abcdefg^Pair-wise comparisons by Bonferroni post-hoc test: ^acd^ *p*<0.0001, ^bef^ *p*<0.001, ^g^ *p*<0.05. (C) Coronal VMB sections of MPTP intoxicated B6 mice reacted with antibodies against CD3, CD4 and CD8 show positive immunostaining of cells with small, round lymphocytic morphology (magnification = 400X).

The presence of NT modifications of α-Syn in draining lymphatic tissue following MPTP-induced nigrostriatal injury, along with evidence of lymphoid-associated APC activation provided support for antigen presentation to T cells and subsequent immune responsiveness. To substantiate this idea, we explored whether an endogenous adaptive immune system was required for MPTP-induced nigrostriatal degeneration. B6 WT mice, B6 SCID mice, and B6 SCID mice reconstituted with 10^8^ B6 WT splenocytes (SPC) (RCS-SCID) were treated with PBS or a chronic MPTP regimen. Mice were sacrificed at 21 days after the last MPTP injection, and VMB sections immunostained for tyrosine hydroxylase (TH), the rate-limiting enzyme in dopamine synthesis (Figure 2A, left panels). The numbers of TH^+^ neurons in the SN showed a 33% reduction in WT B6 animals that received MPTP compared to those that received PBS (Figure 2B). No significant difference in the numbers of TH^+^ neurons was observed in MPTP-treated SCID mice compared to SCID mice that received PBS (Figure 2B). In contrast, immune reconstituted SCID mice (RCS-SCID) treated with MPTP showed significantly fewer TH^+^ neurons compared to the SCID MPTP group (Figure 2A and 2B). To validate the reconstitution of RCS-SCID mice, spleens were immunostained for CD3^+^ T cell distribution (Figure 2A, right panels). T cell repopulation was confirmed by the presence of CD3^+^ T cells in the periarteriolar lymphoid sheath of RCS-SCID mouse spleens (Figure 2A),

VMB and cerebellum control sections of WT, SCID, and RCS-SCID mice treated with MPTP were immunostained for T cells using antibodies against CD3, CD4, and CD8. CD3 immunostaining of MPTP-treated B6 mice demonstrated CD3^+^ cells in the VMB beginning at day 0, present at day 4 after MPTP intoxication (Figure 2C) that persisted to day 14. Both CD4^+^ and CD8^+^ subpopulations were also present in VMB of only MPTP-treated animals at 4 and 14 days (Figure 2C). No T cell accumulation was observed in PBS or MPTP-treated SCID mice at any time point (data not shown), whereas CD3^+^ T cell accumulations in VMB of RCS-SCID mice after MPTP-treatment were identified. Cerebellar tissue of MPTP animals had ≤1 CD3^+^ T cell per high power field examined present suggesting specific cell entry into affected regions. Taken together, these data support the occurrence of an adaptive immune response triggered by modified CNS antigens that modulates the vulnerability of the dopaminergic neurons to MPTP through the migration of T cells into the CNS.

### Prediction of Mouse N-α-Syn Specific T Cell Epitopes

To test the probability of N-α-Syn induced adaptive immune responses, we compared the numbers of predicted α-Syn specific T cell epitopes with the propensity to bind class I MHC grooves [48] for the murine MHC haplotypes, H-2^k^ and H-2^b^ (see Table 2). However, since the last 40 aa of the mouse α-Syn contains 4 Tyr residues available for nitration, our analysis focused on this C-terminal α-Syn fragment. Table 2 shows that H-2K^k^ epitopes have a superior ability to bind with high and intermediate affinity α-Syn-derived 8-11-meric peptide fragments derived from the whole α-Syn molecule or from its C-terminal 38-meric fragment. These data demonstrate significant T cell induction potential. In fact, α-Syn has 100 potential T cell epitopes, 73 of which contain Tyr that were predicted to bind H-2K^k^ molecules, while only 8 and 15 potential epitopes may bind H-2D^b^ and H-2K^b^ molecules, respectively. This was also for nitrated epitopes containing Tyr residue including those with a Tyr residue within the central region of the epitope that presents prominently to the T cell receptor. These epitopes do not contain anchor aa that mediate binding to the MHC groove. Dramatic difference in the number of Tyr-containing T cell epitopes from C-terminal segment of α-Syn predicted to bind H-2K^k^ but not any of H-2^b^ molecules (Table 2, data in brackets) suggests a potential preference for H-2K^k^ versus H-2^b^ mice to induce T cell responses to nitrated C-terminal fragments of α-Syn. These fragments, if facing T cell receptors, have greater chances of inducing MHC class I-restricted CD8^+^ T cells due to lack of negative selection against N-α-Syn epitopes in the embryonic thymus. Further epitope prediction analysis revealed a number of 15-meric epitopes, including Tyr-containing peptides from C-terminal, that can bind with increased affinity class II MHC groove, thus increasing the propensity of inducing MHC class II-restricted CD4^+^ T cells specific for Tyr-containing α-Syn C-terminal fragments. Therefore, mice expressing MHC class I and II molecules of H-2^k^ haplotypes are capable of generating immune responses to NT-modified α-Syn. Interestingly, that α-Syn C-terminal fragment contains several Tyr-containing peptides with predicted significant binding affinity for IA^k^ and IA^b^ MHC molecules (18 and 14 epitopes, respectively) suggests a significant potential for CD4^+^ T cells of mice expressing IA^k^ or IA^b^ to respond to nitrated epitopes from α-Syn C-terminal.

**Table 2:** Numbers of putative α-Syn epitopes for presentation to T cell receptors predicted from the binding potential of MHC class I and II molecules for the aa sequence of α-Syn.

Predicted binding of epitopes	Number of predicted epitopes for:					
	^a^MHC class I			^b^MHC class II		
	K^k^	D^b^	K^b^	IA^k^	IE^k^	IA^b^
All	143 (100)	72 (1)	73 (13)	116 (32)	59 (8)	129 (32)
High	29 (26)	1 (0)	1 (1)	19 (12)	0 (0)	2 (2)
Intermediate	71 (58)	7 (0)	14 (6)	55 (13)	6 (1)	70 (16)
Low	43 (16)	64 (1)	58 (6)	42 (7)	53 (8)	57(14)
Containing Tyr	101 (77)	50 (1)	48 (8)	34 (23)	8 (5)	33 (24)
High	24 (21)	1 (0)	1 (1)	9(10)	0 (0)	2 (2)
Intermediate	49 (43)	7(0)	12 (4)	19 (8)	0 (0)	16 (12)
Low	28 (13)	42 (1)	35 (3)	6 (5)	8 (5)	15 (10)

Number of predicted T-cell epitopes from the 38-mer C-terminal fragment of α-Syn are in brackets. ^a^For class I MHC binders three following grades for scoring included: low immunogenic epitopes, scores <−3; mild immunogenic epitopes, scores >−3 but <−2; high immunogenic, scores >−2; all computations were done using the Immune Epitope Database and Analysis Resource (IEDB) (http://immuneepitope.org) and integrative epitope prediction tool [proteasomes cleavage, Transporter associated with Antigen Processing (TAP) binding, processing and MHC binding]. ^b^For class II MHC binders, scoring grades were based on predicted IC_50_ values and were: low (1000 nM–5000 nM), intermediate (200–1000 nM) and high (<200 nM) for groove binding prediction by MHCPred. This prediction algorithm considers peptides with predicted IC50 >5000 nM as non-binders [86], [87].

### Purification and Nitration of Recombinant α-Syn

**Figure 3:**
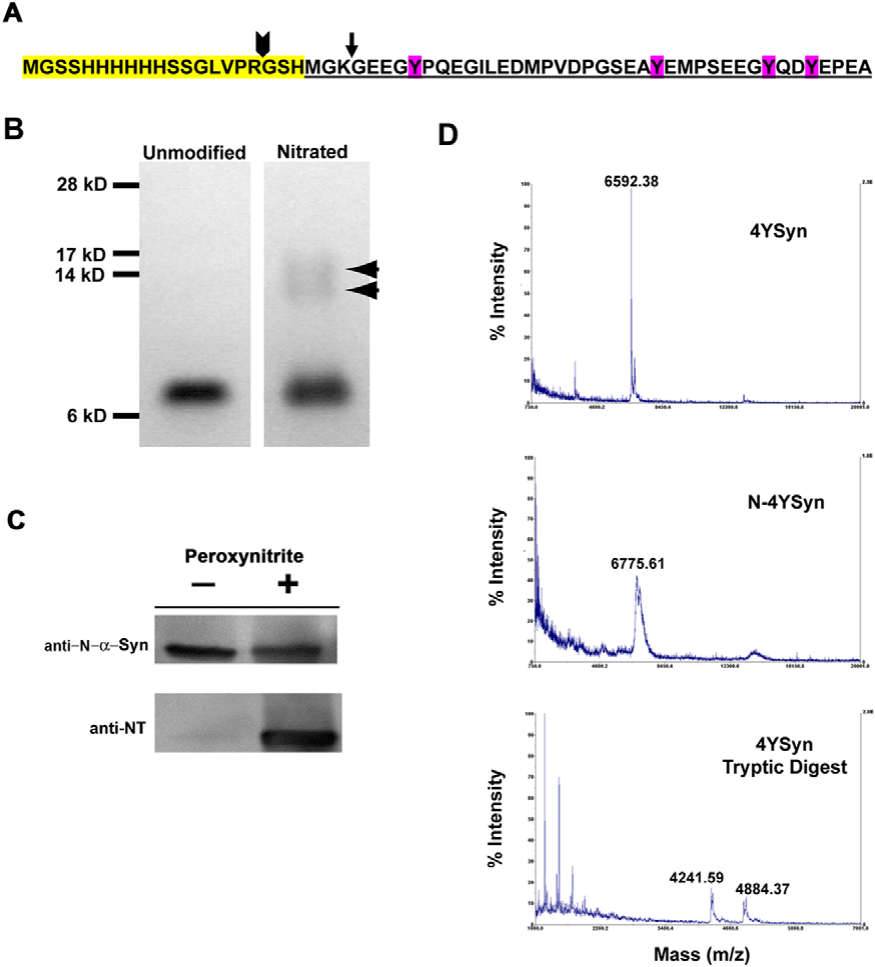
Characterization of purified and nitrated recombinant 4YSyn. (A) Primary aa sequence of His-tagged 4YSyn peptide. The His-Tag sequence is highlighted in yellow. The sequence of 4YSyn (Syn_100–140_) is shown underlined with 4 Tyr residues (magenta) as potent sites for nitration. Trypsin cleavage sites at Arg (arrowhead) and Lys (arrow) are shown. (B) Purified 4YSyn (lane 1) and N-4YSyn following nitration with peroxynitrite (lane 2) fractionated on a 10–20% polyacrylamide gel and visualized using silver stain. Covalently cross-linked oligomers are indicated by arrowheads. (C) Western blot confirmation of purified 4YSyn and its associated NT modifications following peroxynitrite treatment. (D) MALDI-TOF spectra of purified 4YSyn (top panel), N-4YSyn (middle panel), and 4YSyn after tryptic digest (lower panel).

Based on our findings, we hypothesized that in PD, NT modifications of α-Syn could be a key step converting the endogenous protein to an immunogen. Here, we used the C-terminal 40 aa α-Syn fragment (4YSyn) as it contains all four tyrosine residues that are nitrated, thus limiting the possible specificities of epitopes capable of generating an immune response. For this, the mouse cDNA encoding the final 40 aa was cloned into the bacterial pET-28a His-tag expression vector and recombinant protein expressed in BL21 *E. coli* following isopropyl-β-D-thiogalactopyranoside (IPTG) induction. Expression of the recombinant protein exhibited no apparent toxicity to the bacterial expression system. Affinity-purified 4YSyn peptide from *E. coli* lysates was detected as a prominent single band using silver staining on 12% polyacrylamide gel (Figure 3B) and by Western blot using a polyclonal antibody raised against aa 120–140 of α-Syn (Figure 3C). Reverse-phase high performance liquid chromatography (RP-HPLC) analysis of isolated 4YSyn products demonstrated purities equal to or in excess of 97%. NT modifications of 4YSyn peptide (N-4YSyn) after peroxynitrite nitration was confirmed by Western blot using mouse monoclonal anti-NT antibody (Figure 3C).

Homogeneity of purified 4YSyn and its modified forms (aggregated and nitrated) was assessed based on: 1D SDS-PAGE (Figure 3B), Western blot (Figure 3C), and matrix-assisted laser desorption ionization-time of flight (MALDI-TOF) mass spectrometry (Figure 3D). The predominant peak for 4YSyn by MALDI-TOF analysis was 6592 m/z, which corresponded to the 6718 expected mass of purified recombinant α-Syn within <2% mass accuracy (Figure 3D and Table 3). To provide proof that the mass discrepancy originated from recombination errors within the His-tag region obtained during protein purification, but not within the biologically active portion of the molecule, we digested the recombinant 4YSyn protein with trypsin and measured masses of resulting fragments using MALDI-TOF. The observed masses of the generated fragments were Arg-cleaved 4YSyn and Lys-cleaved 4YSyn. These corresponded to the expected masses with 0.07% of mass accuracy. Next, we compared the mass of native 4YSyn to N-4YSyn. The oxidized peptide or its trypsin cleaved fragments revealed a 184 D mass increase that is analogous to the expected mass of 4 nitro groups corresponding to 4 NT- residues (Figure 3A and 3D). Based on these observations, we concluded that reaction of 4YSyn with peroxynitrite, under the conditions used in this study, efficiently nitrated all four available Tyr residues in 4YSyn.

**Table 3:** Theoretical and Observed Masses of 4YSyn, N-4YSyn and Tryptic Digest Fragments.

Peptide	Theoretical Mass (D)	Observed Mass (D)
His-Tagged 4YSyn	6718	6592
His-Tagged N-4YSyn	6902	6775
Arg Cleaved 4YSyn	4836	4833
Lys Cleaved 4YSyn (K)	4238	4241
Arg Cleaved N-4YSyn	5020	5073
Lys Cleaved N-4YSyn	4422	

### N-4YSyn Induces Specific Immune Responses in B10.BR Mice

**Figure 4:**
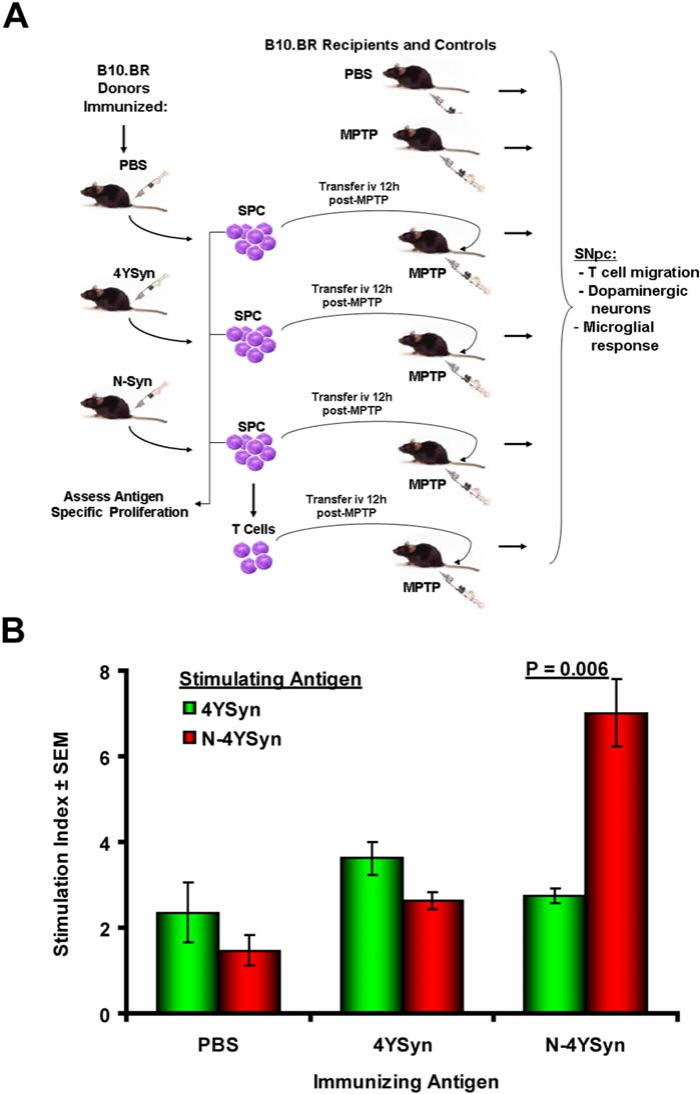
Experimental protocol for adoptive transfer and lymphocyte proliferation assessment of donor SPC in B10.BR mice. (A) B10.BR (H-2^K^) mice were immunized with PBS, 50 µg 4YSyn, or 50 µg N-4YSyn emulsified in CFA. Mice were boosted 14 days later with PBS or their respective antigens in IFA. After 5 days, donor mice were sacrificed and single cell suspensions were prepared from the draining inguinal lymph nodes and spleen, and T cells were enriched by negative selection. Twelve hours after the final MPTP injection, 5×10^7^ donor immune SPC or 2.5×10^7^ T cells were adoptively transferred to MPTP-treated recipient mice. SPC were evaluated for antigen specificity prior to adoptive transfer by lymphocyte proliferation assays. SN of recipients were evaluated after 28 days of MPTP treatment for migration of T cells, survival of dopaminergic neurons, and reactive microglia. (B). SPC were tested for antigen specific proliferation by culturing in the presence of media alone or media containing 3 µg/ml of immunizing antigens for 5 days and using standard ^3^H-thymidine incorporation assays.

To test our predictions of immune responses to N-α-Syn, B10.BR (H-2^k^) mice were immunized with N-4YSyn, 4YSyn, or PBS each emulsified in complete Freund's adjuvant (CFA) (Figure 4A). Fourteen days following the initial immunization, mice were boosted with their respective immunogens emulsified in incomplete Freund's adjuvant (IFA). Five days later, mice were sacrificed and SPC were tested for antigen-specific T cell proliferative responses to N-4YSyn or 4YSyn. Stimulation with 4YSyn yielded no significant immune responses regardless of whether mice were immunized with adjuvant containing PBS, 4YSyn or N-4YSyn (Figure 4B). In contrast, significant proliferative responses were afforded from SPC of mice immunized with N-4YSyn and challenged *in vitro* with N-4YSyn, but not 4YSyn. Moreover, N-4YSyn stimulated SPC from mice immunized with adjuvant containing 4YSyn or PBS failed to induce significant proliferative responses. These data indicate that immunization with N-4YSyn, but not 4YSyn is capable of inducing antigen specific immune responses to NT-modified CNS antigens.

### Adoptive Transfer of N-4YSyn SPC and T Cells Exacerbates MPTP-induced Microglial Activation and Dopaminergic Neuronal Death

**Figure 5:**
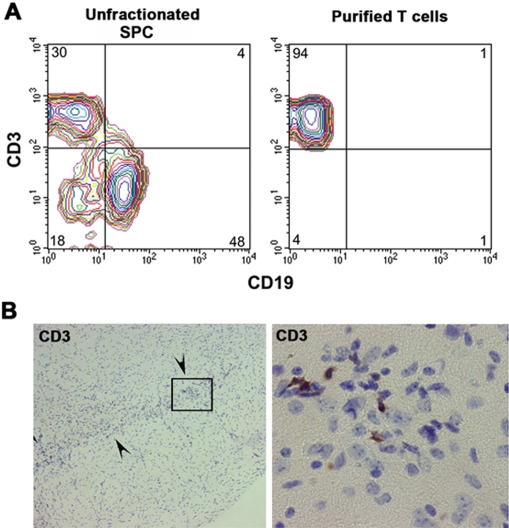
Adoptive transfer of SPC and purified T cells from N-4YSyn vaccinated B10.BR donors leads to infiltration of T cells in the SNpc of MPTP mice on day 2. (A) Frequency of CD3^+^ T cells and CD19^+^ B cells before and after enrichment of T cells. Population of enriched T-cells was 94% CD3^+^ prior to adoptive transfer to B10.BR mice. (B) Sections throughout the SNpc were immunostained for CD3 and counterstained with thionin. Clusters of CD3^+^ cells are observed within the SNpc (arrowheads) as seen at 100× magnification (left). Magnification (600X) of boxed area (left panel) is shown (right panel). CD3^+^ cells are small and round exhibiting lymphocyte morphology.

In light of the fact that modified α-Syn is capable of evading tolerance and inducing reactive T cells, we next tested whether modified α-Syn-activated T cells could exacerbate MPTP-induced dopaminergic neurodegeneration. The experimental scheme for adoptive transfer of SPC or purified T cells from immunized animals is outlined in Figure 4A. For these studies, B10.BR (H-2^k^) donor mice were immunized and boosted with N-4YSyn or 4YSyn, and SPC were adoptively transferred to MPTP-treated syngeneic recipients. To delineate effects due specifically to T cells, CD3^+^ T cells were enriched by negative selection and transferred to an additional group of MPTP-treated animals. Flow cytometric analysis showed that the enriched population from N-4YSyn mice was 94% CD3^+^ T cells (Figure 5A). Adoptive transfer of purified T cells from N-4YSyn immunized donors to MPTP intoxicated mice revealed CD3^+^ T cell infiltrates in the SNpc on day 2 after MPTP treatment (Figure 5B).

**Figure 6:**
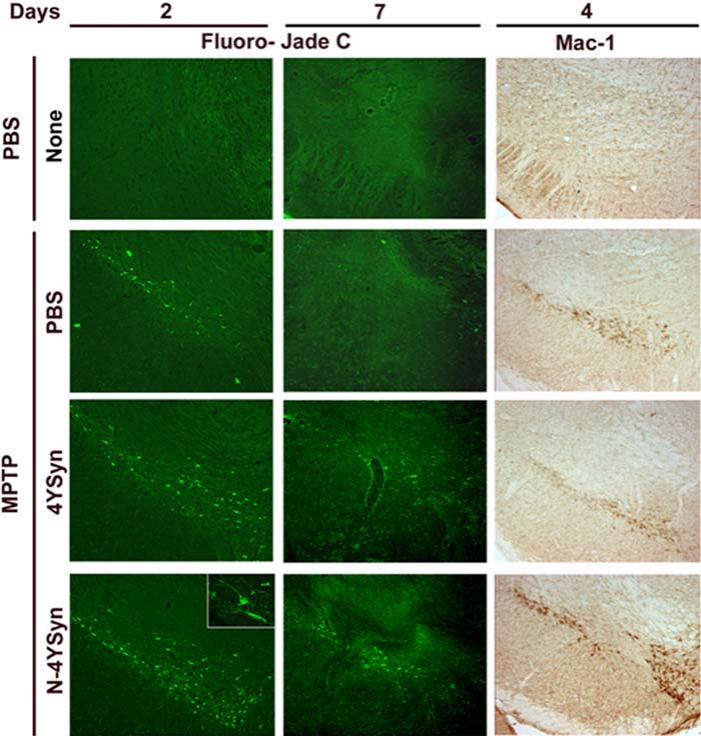
SPC from N-4YSyn immunized B10.BR mice exacerbate MPTP-induced dopaminergic neurodegeneration and induce microglial responses in the SNpc. Photomicrographs from VMB sections stained with Fluoro-Jade C (left and middle panels) and Mac-1 antibody (right panels). PBS controls (PBS/none) exhibit an absence of Fluoro-Jade C stained dead neurons on days 2 and 7, and only faint Mac-1 immunoreactivity on day 4 post-treatment. In MPTP-treated mice that received SPC from PBS/adjuvant treated donors (MPTP/PBS), Fluoro-Jade C stained neurons are evident at day 2, but not detectable by day 7. MPTP-treated mice that received SPC from 4YSyn immunized donors (MPTP/4YSyn), also exhibits dead fluorescent neurons by day 2 comparable to the MPTP/PBS control group, and only rare degenerating neurons are visible by day 7. Mac-1 immunoreactivity in those mice is comparably resolved to levels seen in MPTP/PBS control group. SPC transfers from N-4YSyn immunized donors to MPTP-treated mice (MPTP/N-4YSyn) induced a robust and prolonged microglial response, conspicuously enhanced when compared to MPTP/PBS-treated controls, with concomitant neuronal death still evident by Fluoro-Jade C staining at day 7.

MPTP treated mice showed fluorescent neurons within the SN using the degenerating cell marker Fluoro-Jade C by day 2, but not by day 7 (Figure 6, left and middle panels) confirming previous kinetic data regarding MPTP-induced nigral neuronal death obtained by silver staining techniques [49]. MPTP treated mice that received immune cells from N-4YSyn immune mice showed more Fluoro-Jade C stained neurons within the SN by day 2 than MPTP-intoxication alone, and, in contrast to the latter, Fluoro-Jade C stained neurons within the SN by day 7 as well. In the PBS control group, no Fluoro-Jade C stained neurons were observed at any time point. Following MPTP administration, microgliosis is striking and immediate. Our initial time course studies are in line with these findings and show that the microgliosis and dopaminergic neurodegeneration in B10.BR mice are virtually resolved respectively by days 4 and 7 post-MPTP injection (Figure 6, right and middle panels, respectively). However, adoptive transfer of SPC from B10.BR mice, regardless of immunization protocol, was associated with a persistent microglial response, as evidenced by quantitative morphology with Mac-1 immunostaining (Figure 6, right panel). Counts of Mac-1^+^ microglia were greatest (p<0.0001) in MPTP mice treated with SPC from N-4YSyn immunized mice [84.1±7.0/mm^2 ^(mean±SEM)] compared to those from mice treated with MPTP and SPC from 4YSyn immunized mice (26.9±3.5/mm^2^), MPTP alone (27.7±3.2/mm^2^), or PBS (0.7±0.3/mm^2^). These data suggest that the adaptive immune components of H-2^k^ mice following MPTP administration contribute to the neuroinflammatory phenotype seen in these animals.

**Figure 7:**
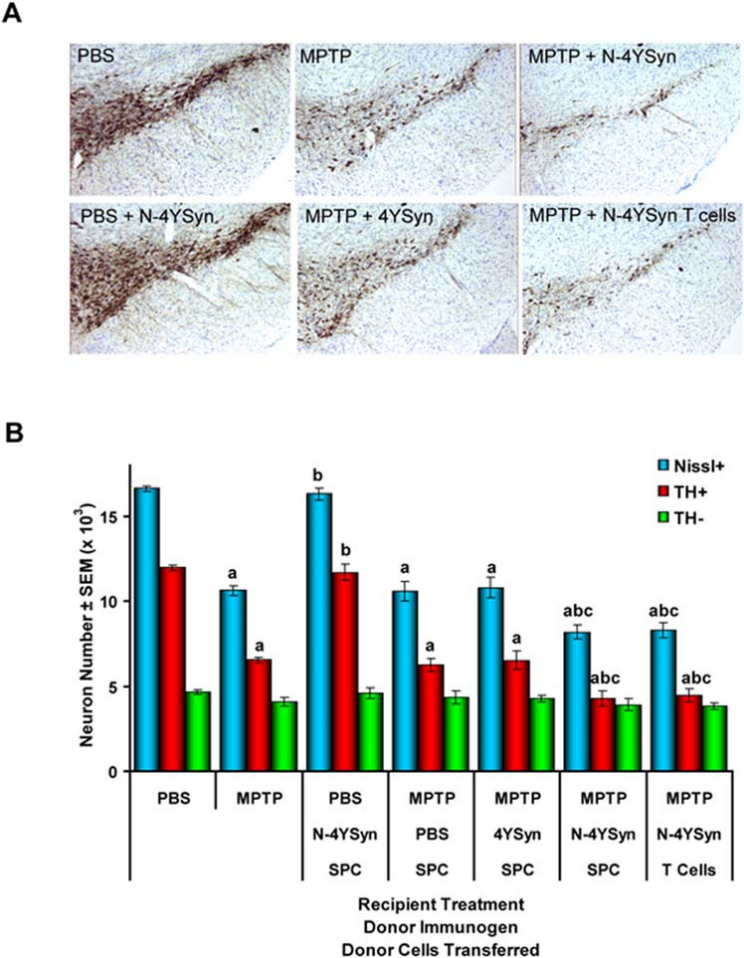
N-4YSyn immunization with adjuvant exacerbates dopaminergic neuronal cell loss in B10.BR mice. (A) All panels show TH-positive neurons in the SN from mice treated with: [top row, L to R] PBS or MPTP alone, MPTP and SPC from N-4YSyn immunized donors (MPTP+N-4YSyn), [bottom row, L to R] PBS and SPC from N-4YSyn immunized donors (PBS+N-4YSyn), MPTP and SPC from 4YSyn immunized donors (MPTP+4YSyn), and MPTP and T cells from N-4YSyn immunized donors (MPTP+N-4YSyn T Cells). Tissues collected 28 days post-MPTP treatment (B) Counts of nigral Nissl+ (blue bars), TH+ (red bars), and TH- (green bars) neurons on day 28 after MPTP treatment as determined by stereological analysis. Control groups included mice treated with PBS alone (n = 4), MPTP alone (n = 8), and PBS animals that received immune effector SPC from N-4YSyn immunized donor mice PBS/N-4YSyn/SPC (n = 6). Experimental groups included MPTP/PBS/SPC (n = 6), MPTP/4YSyn/SPC (n = 8), MPTP/N-4YSyn/SPC (n = 9), and MPTP mice which received purified T cells from N-4YSyn vaccinated donors. Values are means ± SEM. *p*<0.01 compared to the following treatment groups: ^a^PBS, ^b^MPTP, ^c^MPTP/4YSyn/SPC.

Based on these findings, we next investigated whether the immune response mediated by N-α-Syn affects degeneration of dopaminergic cell bodies in the SNpc. To test this, MPTP-intoxicated B10.BR mice received 5×10^7^ donor SPC from mice immunized with PBS, 4YSyn, or N-4YSyn, or 2.5×10^7^ enriched T cells from 4YSyn-immunized donors. PBS- and MPTP-treated mice that did not receive cells served as controls for no neuronal loss and loss attributable to MPTP treatment alone, respectively. PBS-treated mice that received N-4YSyn immunized donor SPC served as additional controls. VMB sections were obtained from mice at 2, 7, and 28 days following MPTP treatment and immunostained for TH (Figure 7A). Stereological analysis showed that MPTP induced a 45% reduction of SN TH^+^ neurons compared to PBS controls (Figure 7B). Similar results were observed in MPTP-injected mice that received SPC from PBS or 4YSyn immune donors (MPTP/PBS/SPC and MPTP/4YSyn/SPC, respectively). In contrast, recipients that received immune N-4YSyn SPC (MPTP/N-4YSyn/SPC) or N-4YSyn T cells (MPTP/N-4YSyn/T Cells) exhibited significantly greater reductions of SNpc TH^+^ neurons (64 and 63%, respectively) compared to all other MPTP-treated animals (Figure 7B). PBS-treated mice that received immune SPC from N-4YSyn immunized donors showed no change in TH^+^ neuron numbers, demonstrating the necessity for an initiating neuronal insult. Significant effects from any treatment were not observed among the numbers of non-dopaminergic neurons (Nissl+TH-). Correlation analysis of total Nissl+ neurons compared to TH+ and TH- neurons demonstrated that the number of total neurons strongly correlated with numbers of TH+ neurons (r = 0.981, p<0.0001) compared to numbers of TH- neurons (r = 0.522, p = 0.004). This confirmed that differences in TH+ neuron counts are due to differences in numbers of structurally intact neurons and eliminates the possibility that differences resulted from the down-regulation of TH itself. Thus, these data demonstrate that adaptive immune responses against the nitrated form of α-Syn exacerbated MPTP-induced nigrostriatal degeneration.

**Figure 8:**
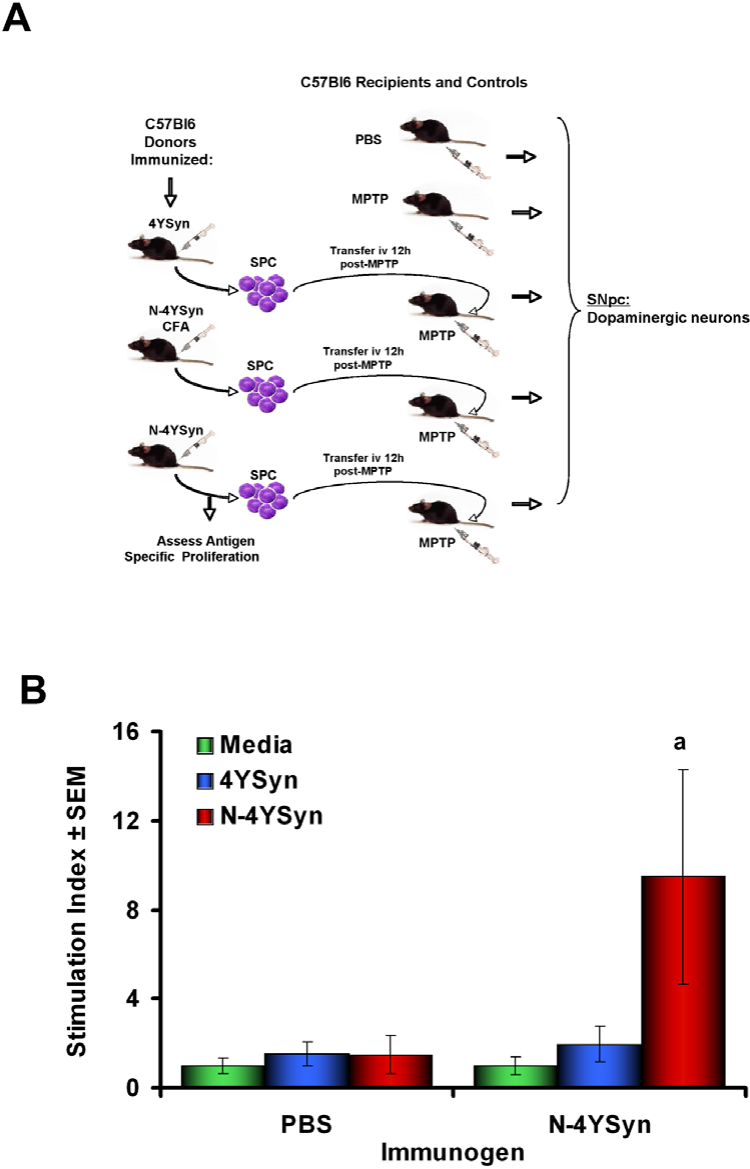
Scheme for immunization, lymphocyte proliferation assessment, and adoptive transfer of donor SPC in B6 mice. (A) B6 (H-2^b^) mice were immunized with 10 µg 4YSyn in PBS, 50 µg N-4YSyn in CFA, 10 µg N-4YSyn in PBS or PBS in CFA. Mice were boosted 14 days later with their respective antigens as formulated previously with or without IFA. After 5 days, single lymphoid cell suspensions were prepared and assessed for antigen-specific responses in standard lymphocyte proliferation assays. Single cell suspensions were pooled and adoptively transferred to MPTP-treated syngeneic recipients 12 hrs after the final MPTP injection. 5×10^7^ donor immune SPC were adoptively transferred to MPTP-treated recipient mice. Survival of dopaminergic neurons in the SN of recipient mice were evaluated after 7 days. (B) Antigen specific proliferation of SPC from B6 (H-2^b^) mice (n = 5/group) immunized with PBS/CFA or N-4YSyn/CFA, and cultured for 5 days in media alone (green bars) or in the presence 1 µg/ml of 4YSyn (blue bars) or N-4YSyn (red bars). Cultures were pulsed for 18 hrs, cells harvested and ^3^H-thymidine incorporation counted by β-scintillation spectrometry. Values represent mean stimulation indices±SEM and analyzed by ANOVA and Bonferroni post-hoc tests. ^a^p = 0.0478.

Based on MHC binding affinity algorithms, mice expressing H-2^b^ were predicted to respond poorly to α-Syn epitopes; yet 21 days after chronic MPTP-intoxication, B6 mice that express the H-2^b^ haplotype yielded significant antibody responses to N-α-Syn. This suggested that mice expressing H-2^b^ have the potential to develop immune responses to N-α-Syn that may affect disease progression. To assess that possibility, we immunized and boosted B6 (H-2^b^) mice with 4YSyn or N-4YSyn either in PBS or emulsified in adjuvant (Figure 8A). Five days after the final boost SPC were harvested, assessed for antigen specific lymphocyte proliferation, and adoptively transferred to MPTP-intoxicated recipients. Stimulation of SPC from PBS-treated controls indicated that neither 4YSyn nor N-4YSyn induced significant proliferation above medium background levels (Figure 8B). In contrast, stimulation of SPC from N-4YSyn/PBS immunized mice with N-4YSyn induced a significant lymphocyte proliferative response indicating that immunization with N-4YSyn/PBS in the absence of adjuvant is capable of inducing an antigen specific adaptive immunity.

**Figure 9:**
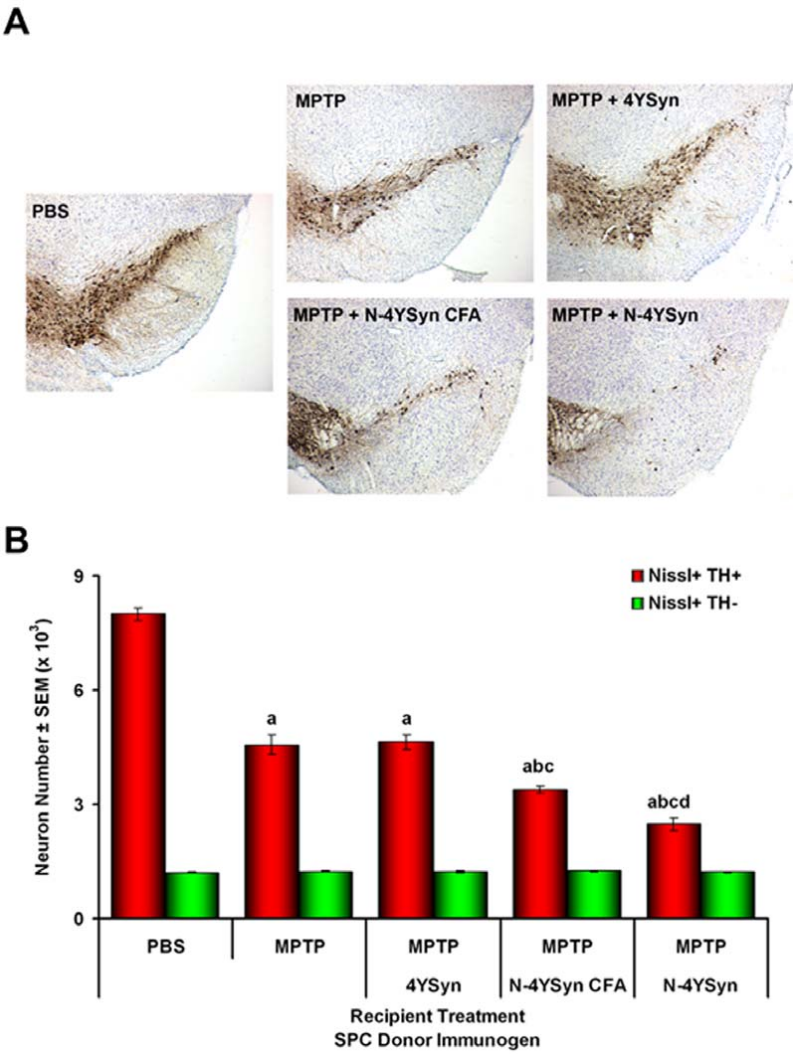
Lymphocytes from N-4YSyn immunization exacerbate nigral dopaminergic neuronal loss in B6 mice. (A) All panels show TH^+^ neurons in the SN from mice treated with PBS or MPTP alone, MPTP and SPC from 4YSyn immunized donors (MPTP+4YSyn), MPTP and SPC from N-4YSyn immunized donors (MPTP+N-4YSyn) and lastly, MPTP and SPC from N-4YSyn+CFA immunized donors (MPTP+N-4YSyn CFA). (B) Counts of nigral TH^+^ and TH^−^ neurons on day 7 after MPTP treatment. Experimental groups included mice treated with PBS alone (n = 7), MPTP alone (n = 7), MPTP/4YSyn (n = 7), MPTP/N-4YSyn/CFA (n = 6) and MPTP/N-4YSyn (n = 6). Values are means ± SEM. Analysis by ANOVA with Bonferroni *post-hoc* tests indicated ^a^p<0.0001 compared to PBS control: ^b^p<0.001 compared to MPTP group; ^c^p<0.001 compared to MPTP/4YSyn group; and ^d^p<0.03 compared to MPTP/4YSynCFA SPC.

TH stained sections of VMB from MPTP-intoxicated mice that received SPC from donors immunized with N-4YSyn either in PBS excipient or adjuvant showed significant dopaminergic neuronal losses compared to MPTP-treated mice or those that received SPC from 4YSyn immunized mice (Figure 9A), suggesting 4YSyn immunization increased the MPTP-induced lesion. Next, we assessed the sections by stereological analysis to obtain estimates of dopaminergic neuronal survival and loss after treatment compared to PBS-treated mice. MPTP-intoxication of B6 mice induced 43% loss of TH+ nigral neurons compared to PBS-treated controls (Figure 9B). Numbers of dopaminergic neurons from MPTP mice treated with SPC from 4YSyn immunized mice were not significantly different compared to MPTP-treated mice and showed a similar 42% loss of neurons. However, adoptive transfer of SPC from N-4YSyn/Adjuvant immune donors significantly increased MPTP-induced dopaminergic neuron loss to 58%. Interestingly, SPC from mice immunized with N-4YSyn without adjuvant induced a 69% loss of nigral TH+ neurons after MPTP intoxication, which was significantly greater than losses due to SPC from 4YSyn- or N-4YSyn/Adjuvant immunized donors. Neither MPTP treatment nor adoptive transfer of immune SPC significantly affected numbers of non-dopaminergic neurons. Thus, taken together these data demonstrate that N-α-Syn, but not unmodified α-Syn, has the capacity to induce specific immune responses by which exacerbates neuronal loss in the context of dopaminergic neurodegeneration. Moreover, these results in H-2^b^ mice, predicted to provide poor immune response, suggests that epitopes modified by inflammatory processes may function unlike their tolerated unmodified self-analogues to induce immune responses to levels sufficient to alter disease progression.

### Nitrated α-Syn Inhibits Proliferation of Anti-CD3 Activated T Cells

**Figure 10:**
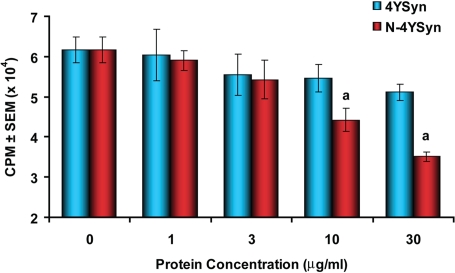
N-4YSyn-mediated inhibition of T cell proliferation. Proliferative responses of anti-CD3 stimulated T cells from naïve B6 mice in presence of graded concentrations of 4YSyn or N-4YSyn (1, 3, 10, 30 µg/ml) or in media alone (0 µg/ml). T cells were cultured for 72 hrs and pulsed with ^3^H-thymidine for the final 18 hrs of culture. Harvested cells were counted for ^3^H-thymidine uptake by β-scintillation spectrometry and proliferation was expressed as mean counts per min (CPM)±SEM for quadruplicate samples and evaluated by ANOVA with Bonferroni *post-hoc* tests. ^a^p<0.01 compared with T cells stimulated with anti-CD3 and cultured in media alone.

We observed that antigen specific proliferative responses were inhibited by N-4YSyn in a dose dependent manner (r^2^ = 0.96, p = 0.002). Significant inhibition was not seen by 4YSyn (r^2^ = 0.53, p = 0.273). To rule out an antigen specific suppressive effect, purified T cells obtained from naïve mice were stimulated with anti-CD3 for 24 hrs in media or in the presence of 4YSyn or N-4YSyn at concentrations of 1, 3, 10 and 30 µg/ml. N-4YSyn inhibited proliferation of anti-CD3 stimulated T cells in a dose dependent fashion (r^r^ = 0.6803, p<0.0001). A significant inhibition of 31 and 47% was observed when stimulated T cells were co-cultured at N-4YSyn concentrations of 10 and 30 µg/ml (Figure 10). In contrast, proliferation of anti-CD3 stimulated T cells was not inhibited by 4YSyn (p = 0.435) or by increasing 4YSyn concentration (r^2^ = 0.1554, p = 0.0854). Moreover, the inverse effect on anti-CD3 activated T cells with increasing N-4YSyn concentration was greater (p = 0.016) compared to that induced by 4YSyn. *Second*, to assess whether this effect was due to a cytotoxic mechanism, anti-CD3 stimulated T cells were stained for the membrane impermeant DNA dye, propidium iodide (PI) that is excluded from intact, living cells, and analyzed by flow cytometry. T cells cultured in the presence of N-4YSyn exhibited a dose-dependent increase in the mean fluorescent intensity (MFI) of PI (r^2^ = 0.9261, p = 0.0008) and in the percentage of PI^+^ dead T cells (r^2^ = 0.9743, p = 0.0018) (Table 4). In comparison, percentages of PI stained dead T cells cultured with 4YSyn and the PI MFI were not different from the basal level in absence of either of the peptides and did not change with increasing 4YSyn concentration (r^2^ = 0.5983, p = 0.1249, and r^2^ = 0.5016, p = 0.1807, respectively). Taken together, increasing N-4YSyn concentrations strongly correlated with inhibition of T cell proliferation, percent PI^+^ T cells, and increased MFI for PI (r>0.945, p<0.0154 for all comparisons combined). In contrast, no significant correlations were associated with increasing 4YSyn concentrations. Thus, cytotoxic interference of immune response induction by N-α-Syn may affect disease progression as these processes are shared by both effector and regulatory T cells.

**Table 4:** N-4YSyn-induced cytotoxicity of stimulated T cells.

aT Cell Treatment	Conc. µg/ml	bPercent PI^+^ T Cells	cMFI	dD	eIndex of Similarity
Medium		5.7	33.0	*	0
4YSyn	1	4.9	32.2	0.08	3.8
	3	6	34.1	0.08	4.2
	10	4.7	25.2	0.1	5.1
	30	4.2	26.8	0.16	8.1
N-4YSyn	1	15.9	42.8	0.31	15.7
	3	17.9	58.7	0.33	15.6
	10	47.8	71.6	0.64	30.9
	30	91.3	106.1	0.87	43.4

a Anti-CD3 stimulated T cells were cultured for 24 hrs in media alone or in the presence of different concentrations of 4YSyn or N-4YSyn, stained with 2 µg/ml of the vital dye, PI, washed, and assessed by flow cytometric analysis for uptake of PI.

b Percentage of T cells susceptible to PI permeation as determined by flow cytometric analysis.

c Mean fluorescence intensity of PI-stained T cells.

d D statistic at α = 0.001 of Kolmogorov-Smirnov (K-S) analysis for fluorescence intensities of PI-stained T cells as the sigmoidal function of the accumulated cell frequency curve and fluorescence intensity (channel number) compared to the curve of PI-stained T cells from the medium control (asterisk) as computed by Cellquest software (BD Biosciences) [88].

e Index of similarity = D/[(n_c_+n_t_)/(n_c_·n_t_)]^1/2^, where n_c_ and n_t_ are the number of events in cell frequency curves for medium control (c) and test substance (t), respectively (BD Biosciences) [88]. An index of similarity = 0 indicates the curves are identical.

### N-4YSyn-Stimulated Immune SPC Enhance Dopaminergic Cell Death

**Figure 11:**
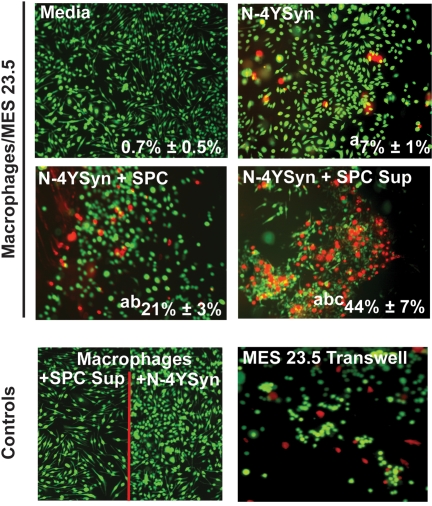
N-4YSyn activated immune SPC induces macrophage-mediated dopaminergic cell death. Representative fluorescence photomicrographs are shown of live (green) and dead (red) cells from 24 hr macrophage/MES 23.5 co-cultures in the presence of media alone, N-4YSyn, N-4YSyn and antigen-stimulated SPC of N-4YSyn-immunized mice (N-4YSyn+SPC), or N-4YSyn and the supernatants from antigen-stimulated SPC of N-4YSyn-immunized mice (N-4YSyn+SPC Sup). Antigen-stimulated SPC from N-4YSyn immune mice were induced *in vitro* with N-4YSyn, and cells and supernatants for use in the assay were harvested after 5 days of culture. Controls included macrophages cultured in the presence of SPC supernatants from antigen-stimulated SPC of N-4YSyn-immunized mice (Macrophages+SPC Sup); macrophages cultured in the presence of N-4YSyn alone (Macrophages+N4YSyn); and transwell cultures of plated MES 23.5 cells and macrophages in the transwell stimulated with N-4YSyn. Frequencies (±SEM) of dead (red) cells for 4-8 fields/assay are provided in the lower right corner of each panel. Differences in the mean frequencies of dead cells were evaluated by ANOVA and Bonferroni *post-hoc* tests. p<0.01 compared to cultures treated with ^a^Media, ^b^N-4YSyn, or ^c^N-4YSyn+SPC.

To directly test the neurotoxic capacity of the N-4YSyn immune response, proliferating T cells or supernatants from N-4YSyn stimulated SPC of N-4YSyn immunized mice were assessed in live/dead assays with co-cultures of N-α-Syn activated bone marrow-derived macrophages (BMM) and MES 23.5 cells. Minimal cytotoxicity was afforded from co-cultures of unstimulated BMM/MES 23.5 co-cultures with virtual 100% MES 23.5 cell survival, whereas activation with aggregated N-α-Syn resulted in 7% cell death after 24 hrs (Figure 11). Activated and proliferating T cells from 5 day cultures of antigen-specific stimulation of N-4YSyn immune SPC when added to activated BMM/MES 23.5 cultures induced 21% cell death (N-4YSyn+SPC), while supernatants (Sup) from those SPC cultures resulted in 44% cell death (N-4YSyn+SPC Sup), and no cytotoxicity to activated BMM cultured in the absence of MES 23.5 cells (Macrophages+SPC Sup). In transwell studies wherein N-4YSyn-stimulated BMM was separated from MES 23.5 targets, virtually all cytotoxicity was concentrated among the MES 23.5 cell population (MES 23.5 Transwell) with no cytotoxicity attributed to BMM. T cells isolated from N-4YSyn-immunized donors and re-stimulated in vitro with N-4Ysyn, but not 4YSyn, showed TNF-α levels (26.0±1.2 pg/ml) that were increased (p<0.008) compared to levels from T cells of either PBS or 4YSyn immunized mice which were below the limits of detection (data not shown).

## Discussion

This study provides direct evidence that the adaptive immune system can exacerbate dopaminergic neuronal loss in animal models of PD. Nitrated proteins that drain from the CNS into lymphatics induce macrophage activation and T cell responses. Moreover, we show that these T cell responses to N-α-Syn elicit profound neurodegeneration. Cell damage resulting from N-α-Syn is not limited to brain cells as it affects both T cell function and numbers, and may, in fact, affect regulatory T cell subsets (Reynolds, et al. 2007, in press). Our data, taken together, suggests that an exacerbated immune response induced by N-α-Syn may play a role in PD pathogenesis.

Under homeostatic conditions α-Syn exists in equilibrium between soluble and lipid membrane bound forms. Unmodified soluble α-synuclein retains a native unfolded structure in its soluble state [6], [50] and exhibits an α-helical structure when membrane bound; the latter being less prone to fibrillation and nitrate modifications [7], [51], [52]. However under disease conditions, α-Syn develops a β-pleated sheet configuration permissive for filament formation [53]. Nitrate modifications can prevent α-helical formation, increase β-sheet and oligomer formation, and prolong the intracellular protein half-life by reducing the capacity for proteasomal degradation [14], [16]. Moreover, N-α-Syn can inhibit filament formation [14] resulting in increased formation of oligomeric protofibrils [17], [50]. Such oligomeric protofibrils are cytotoxic through pore-forming activity within the plasma membrane [54]. Likewise, environmental cues are known to be linked to PD [55], [56] and elicit significant effects on intraneuronal concentrations, post-translation modifications, conformational behavior, and aggregation of α-Syn [57]. These observations, taken together, support an environmental role for sporadic PD.

Peripheral immune abnormalities have been widely described in PD including the occurrence of autoantibodies against neuronal structures and high numbers of microglia cells expressing human leukocyte antigen (HLA)-DR in the nigrostriatal system [58], [59]. Disturbed cellular and humoral immune functions in peripheral blood of patients with PD have been also identified and T cell infiltration of diseased CNS tissues have been reported [60], [61]. An elevated T cell population expressing γ/δ T cell receptors and increased IgG levels in CSF to heat shock proteins have been also described in PD [62], [63]. T helper cell analysis has revealed a decreased percentage of CD45RA^+^ “naïve” and an increased percentage of CD45RO^+^ “memory” T cell subsets in CD4^+^ T cell populations in the peripheral blood of patients with PD [61]. Collectively, these studies suggest that perturbations in the adaptive immune system may play a role in the progression of the human disease.

There is mounting evidence that immune abnormalities exist in PD. Increased HLA expression, CNS T cell infiltration [59], IgG1 deposition in Lewy Bodies, and peripheral immune abnormalities have been reported. Moreover, the recently demonstrated significant increase of the DQB1*06 MHC class II allele in idiopathic PD may indicate an association between sporadic PD and the immune system [64]. Le and colleagues reported that immunization of guinea pigs with homogenized MES 23.5 cell extracts in CFA resulted in nigrostriatal injury demonstrating the capability of adaptive immune components to injure dopaminergic neurons [65]. However, this approach used a cell line derived from rat embryonic mesencephalon cells that were fused with mouse neuroblastoma cells as an immunogen in the guinea pig. This experimental strategy precludes the challenge of breaking tolerance to self, the primary hurdle in autoimmune pathology, as mouse or rat proteins would likely be viewed as “foreign” in the guinea pig. Moreover, this work models PD as a primary autoimmune disease, for which little supporting evidence exists, and does not give any indication as to how, or under what circumstances, such autoimmune reactions may occur. Our data provides a rationale and a novel mechanism, which includes immunity to modified self-proteins as a disease modifier, rather than autoimmunity as a primary pathological mechanism. This idea nicely supports existing hypotheses where toxic injury (environmental or endogenous) results in damaged self-proteins capable of immune activation. Adaptive immune responses may play a role in amplifying and sustaining innate immune mechanisms operative in PD. Moreover, this may provide insight as to why antigenically related sites are also affected in PD such as the locus coeruleus, raphe nuclei, and nucleus basalis of Meynert.

Innate neuroinflammatory reactions, primarily associated with activated microglia, significantly contributes to secondary neuronal loss in animal models of PD [66], [67]. Mice intoxicated with an acute regimen of MPTP exhibit a robust inflammatory response, which precedes neuronal loss and resolves by day 4 post-intoxication during which most degeneration takes place. The acute nature of this inflammatory response seen in mice is in striking contrast to idiopathic PD that slowly progresses over a period of years. Unlike the mouse data, humans and non-human primates intoxicated with MPTP have sustained microglial activation [68], [69]. Mechanisms linking innate immune activation and dopaminergic neuronal cell loss are under active study. One potential mechanism for sustaining innate neuroinflammatory reactions includes peripheral activation of the adaptive immune system against CNS antigens originating in damaged tissue. Foreign antigen introduced into CNS compartments, including the anterior chamber of the eye or striatum [70], results in the education of peripheral T cells in the spleen or CLN, respectively, and primarily favors tolerance induction [71]. Interestingly, dendritic cells populate perivascular regions of the CNS in normal physiological and pathological states, and have been proposed to migrate out of the CNS where they may act as APC in secondary lymphoid organs. “New” brain antigens (foreign or modified self) presented to the immune system during CNS inflammatory disease states may not favor tolerance induction. Here, we first detected a NT-modified protein with molecular mass <20 kD within the CLN of MPTP-treated mice, which was not observed in any other lymphoid tissue examined. Later this NT-modified protein was unambiguously identified as α-Syn by IP with N-α-Syn specific antibody and in-gel tryptic digestion with protein sequencing by LC MS/MS of the 12–18 kD band after 1D-SDS PAGE. Several lines of evidence support the possibility of drainage of CNS antigens into the CLN. *First*, ovalbumin (OVA) antigen directly injected into the striatum of mice resulted in antigen-specific T cell education exclusively in the deep CLN [72]. *Second*, in our model system, MBP, which is specific to the CNS, was also identified by Western blot at the same time suggesting that brain-derived antigens were draining to the CLN post-MPTP intoxication. *Third*, peroxynitrite favors nitration of Tyr residues on α-Syn [73]. Two different strains of mice, B10.BR (H-2^k^) and B6 (H-2^b^), expressing disparate MHC haplotypes were employed. Predictions of MHC binding affinities for α-Syn T cell epitopes suggested an increased number of peptides with intermediate and high degree binding to class I MHC molecules of H-2^k^, but not H-2^b^. An overall stronger immunogenic binding potential even to MHC class II was predicted for H-2^k^ when compared to H-2^b^. These predictions suggested the 2 strains would exhibit different levels of T cell responses to N-α-Syn with consequential heterogeneous outcomes affecting disease. It is not clear, whether in PD patients the C-terminal fragment of α-Syn, which is nearly identical in mice and human, is nitrated. However, earlier studies demonstrated that monoclonal antibodies to N-α-Syn recognize fragments that include nitrated Tyr^125^ and Tyr^133^ residues within α-Syn C-terminal fragment [74]. Recent studies confirmed preference of NT in close proximity to carboxylates [75]. This is consistent with previous data that promote nitration of Tyr by nearby negative charges [76]. Indeed, within the Tyr-containing 42 aa C-terminal of α-Syn more than 28% (12 aa) are charged negatively (Figure 3A). All together, we posit that monitoring of T cell responses to epitopes predicted in this study may determine the association of neurodegeneration and α-Syn immune responses.

The focus of the current study was to elucidate and characterize new cellular and molecular mechanisms that explain the prolonged neuroinflammation and progressive dopaminergic neurodegeneration in PD as modeled by the administration of MPTP. B10.BR or B6 mice immunized with the unmodified C-terminal tail of α-Syn failed to generate detectable proliferative responses after SPC were stimulated with 4YSyn or N-4YSyn, thus suggesting a degree of tolerance to this self-protein. Conversely, both strains of mice immunized with N-4YSyn developed robust proliferative responses to the modified protein *in vitro*. Interestingly, immune cells that recognized only the modified form did not proliferate in response to the unmodified form suggesting a high degree of specificity in generating adaptive immune responses to the nitrated epitope. Exacerbation of dopaminergic neuronal loss in both MPTP-intoxicated B6 and B10.BR mice that received N-4YSyn immune cells from syngeneic donors was observed in our studies. The loss of dopaminergic neurons within the SN of those recipient mice was significantly greater compared to MPTP-treated animals or those that received SPC from mice immunized with 4YSyn or PBS. Transfer of SPC from mice immunized with unmodified 4YSyn yielded no difference in SN dopaminergic neuronal loss compared to those mice treated with MPTP alone. Importantly, the transfer of purified T cells from N-4YSyn immunized donors exacerbated nigral degeneration in B10.BR mice. Elimination of adjuvant (CFA/IFA) from the immunization strategy for B6 donors and subsequent adoptive transfer of N-4YSyn immune SPC produced the most severe dopaminergic neuronal losses. While behavioral testing of MPTP mice was considered to substantiate dopaminergic neurodegeneration, those were not performed as rodents do not develop typical parkinsonism and behavioral testing in MPTP mice has not proven to be consistently reliable [77], [78].

While the exact phenotype of the T cell response generated in this vaccination strategy is unknown, a proinflammatory T_H_1 response was likely generated and associated with exacerbation of inflammatory responses and neurotoxicity. We also demonstrated *in vitro* that antigen-activated immune responses to N-4YSyn augment N-4YSyn-induced, cell-mediated dopaminergic toxicity with macrophages activated by N-α-Syn, linked to an induction of TNF-α production by stimulated T cells. This suggests a potent mechanism for exacerbation of inflammatory responses and progression of dopaminergic neurodegenerative processes conferred by N-4YSyn specific T cells. These data also emphasize caution in design of vaccine-based therapeutic strategies for PD or other neurodegenerative disorders. Recently, a promising Aβ vaccine strategy was employed in transgenic amyloid precursor protein (APP) mice as a model for Alzheimer's disease [79], [80]. In these studies, immunized APP transgenic animals showed markedly reduced Aβ deposition, preservation of normal neuronal structures, and improved performance in memory and spatial learning tasks. After completing a successful phase I trial with no significant toxicities, phase II trials came to an abrupt end when 15 vaccine recipients experienced meningoencephalitis [81], [82], [83]. Clearly, immune system can generate T cell responses to proteins that are similarly modified as those found in PD but alternative routes of administration may generate entirely different effects on the disease process and as such do not preclude using these proteins as potential vaccine candidates. For example, nasal administration favors the generation of regulatory type T_H_2/T_H_3 responses to antigens [84], [85]. Mucosal vaccination using N-4YSyn may result in dramatically different results than those presented here.

All together, we demonstrated for the first time, that NT α-Syn modifications circumvent immunological tolerance. We posit that these observations are directly relevant to the tempo and progression of PD. Indeed, NT-modifications of α-Syn are present in Lewy bodies during disease and when present in the extracellular spaces of the brain appears in the CNL where a robust adaptive immune response is produced that accelerates nigral dopaminergic neuronal degeneration. Most importantly the abilities of N-α-Syn to evade immunological tolerance provides a novel pathogenic mechanism for PD and new opportunities for therapeutic intervention.
